# Applicability of Exercise and Education Programmes for Knee Osteoarthritis Management to Switzerland

**DOI:** 10.3389/frhs.2021.760814

**Published:** 2021-11-16

**Authors:** Lea Ettlin, Anne-Kathrin Rausch Osthoff, Irina Nast, Karin Niedermann

**Affiliations:** ^1^Schools of Health Professions, Institute of Physiotherapy, Zurich University of Applied Sciences, Winterthur, Switzerland; ^2^Department of Health Sciences and Health Policy, University of Lucerne, Lucerne, Switzerland

**Keywords:** exercise and education programmes, implementation, knee osteoarthritis, RE-AIM, applicability

## Abstract

**Objectives:** The aim of this study was to assess the applicability of six OARSI (Osteoarthritis Research Society International) approved exercise and education programmes for the conservative management of knee osteoarthritis to the Swiss health care system.

**Methods:** The RE-AIM framework was used in this cross-sectional survey study to analyse the characteristics of the six exercise and education programmes. A survey was developed based on the RE-AIM dimensions, “Reach, Effectiveness, Adoption, Implementation, and Maintenance,” for rating the applicability of the programmes (on a scale of 1 = “least applicable” to 10 = “most applicable”). Programme scores of ≥7 indicated applicability to the Swiss health care system. Nine selected physiotherapy experts for knee OA management in Switzerland were invited for the rating.

**Results:** The six programmes were rated by six of the nine invited research experts with mean scores of between 5.9 and 9.45. Four programmes scored 7 or more. These four programmes all included supervised exercise sessions and education with the goal that the participants understand the diagnosis and the management of OA. The two lower rated programmes focused on exercise counselling or weight reduction.

**Conclusion:** The programme with the highest scores consists of exercise and education and scored higher than 7 in all RE-AIM dimensions. Therefore, this programme is most applicable to the Swiss health care system as only a few adaptations would be needed for its successful implementation.

## Introduction

The international clinical guidelines for the management of knee osteoarthritis (OA), which were developed by the OA Research Society International (OARSI), American College of Rheumatology (ACR), and European Alliance of Associations for Rheumatology (EULAR), recommend exercise, education, and weight control, when appropriate, as first-line interventions (1–3). Pharmacological therapy or passive treatment might support first-line interventions (1–3). There is strong evidence that regularly performed exercises for knee OA have a positive effect on pain and joint function (1–5). The long-term goal of these interventions is the enhancement of self-management in people with knee OA. However, clinical practise is often at variance with guidelines (6–10). Because of this, the OA guideline recommendations have already been translated into exercise and education programmes, or models of care, in several countries to make their application feasible and simplify implementation into clinical practise. There are six well-established, OARSI-endorsed programmes that meet the specific needs of their national health care systems (9, 10). These programmes have been successfully applied in real-world settings and have been proven effective (11).

According to national data, knee OA is the most common diagnosis in Swiss hospitals (12). However, both research and observation of clinical practise have shown that the guidelines recommendations are not being systematically applied in knee OA management and exercise and education appear to be underexploited in Switzerland, which results in an evidence-performance gap (13). In accordance with experience from other countries, the implementation of a national exercise and education programme in Switzerland would contribute to overcoming this evidence-performance gap (9, 10). Consequently, a network of physiotherapy experts for knee OA management was formed in 2019 to promote the implementation of an exercise and education programme, with a preference for one that was already established and approved by OARSI. The network included six researchers from three Universities of Applied Sciences in the three national language areas of Switzerland, i.e., German, French, and Italian, two clinical practitioners representing each of two different specialist physiotherapy societies, and one patient representative of the Swiss League Against Rheumatism (SLAR).

The implementation of a structured programme in a health care system is challenging and time-consuming (9, 10, 14, 15). For the successful implementation of a structured programme for knee OA, it must be aligned to the characteristics of the Swiss health care system and meet local needs. Switzerland's health care system is organised at the cantonal level and is decentralised, complex, and has a high level of local autonomy. This structure, along with the fee for service reimbursement (16), represent barriers to the implementation of new care structures and innovations (17). Many aspects (e.g., programme characteristics, provider characteristics, community factors, and health care system structures) influence the implementation process and need to be considered carefully (18). This has proven to be the case for other disease management programmes, such as diabetes (17). An implementation into the Swiss health system requires not only an analysis of the clinical effectiveness of the programme, but also an assessment of the necessary national adaptations. However, Rodriguez and Bernal (19) have stated that in the implementation of evidence-based treatment, the more cultural adaptations of an innovation are made, the higher the risk of compromising the effectiveness of the treatment, i.e., essential components linked to positive outcomes are changed and may compromise the effectiveness of the treatment (20, 21). Therefore, the process for selecting a programme applicable to Switzerland should be: 1. Identification of the programme that best meets the needs and requirements of the Swiss health care system, and 2. Application of the rule “The less adaptations, the better.”

The aim of this study was to analyse the six OARSI-approved exercise and education programmes, using the RE-AIM framework, to assess their applicability for implementation in the Swiss health care system.

## Methods

### Design

This cross-sectional survey study was based on a secondary analysis of the six OARSI-approved exercise and education programmes, using the RE-AIM framework (15) to rate the applicability of the programmes for implementation in Switzerland.

### Participants

All members of the above-mentioned network of experts in knee OA management were invited to participate in the survey and to rate the applicability of the programmes. They were personally informed of the survey and invited to participate by email, with the survey (Word-document) attached. A follow-up reminder was sent 2 weeks later.

For transparency and consistency of reporting in implementation studies, this study follows the “Standards for Reporting Implementation studies” (StaRI) statement.

### Characteristics of the OARSI-Approved Programmes

The six OARSI-approved programmes are: 1. “Osteoarthritis Chronic Care Program (OACCP) Australia,” 2. “Better management of patients with osteoarthritis (BOA) Sweden,” 3. “Good Life with Osteoarthritis in Denmark (GLA:D^®^),” 4. “Osteoarthritis Healthy Weight For Life (OA HWFL) Australia,” 5. “Amsterdam osteoarthritis cohort (AMSOA) of The Netherlands,” and 6. “Joint Implementation of Osteoarthritis guidelines in the West Midlands” (JIGSAW). The characteristics of the six programmes, evaluated using the RE-AIM dimensions, regarding target population, interventions, duration, primary outcomes, number of patients enrolled, and reimbursement, were extracted from published studies, reports, and the websites of the respective programmes. Table 1 displays a short overview of the programmes, showing the aim of the programme, how many people were enrolled, and how the local reimbursement system was organised.

**Table 1:** Overview of exercise and education programmes.

**Programme**	**Country of origin**	**Aim of programme (published on their website, if available)**	**Number of patients enrolled**	**Reimbursement policies**
BOA (8, 10, 22–24)	Sweden	All patients with OA in Sweden should be offered adequate information and exercise according to the guidelines	Approx. 10,941 patients/year (2008–2020)	Coverage by universal healthcare: 100% coverage with a limited out-of-pocket spending (maximum fee of ~120 USD for outpatient visits during a 12-month period)
OACCP (25–28)	Australia	A pathway to improved care is offered for people who have one of the most common, debilitating, costly and rapidly growing chronic conditions—OA	Approx. 1,250 patients/year (2012–2020)	Coverage by universal healthcare (Medicare): Subsidies of supervised exercise sessions and consultations (assessments) according to management plan with a possible rebate from the private health insurers. Providers set the cost of the program
GLA:D^®^ (7, 29, 30)	Denmark	Implementation of current clinical guidelines for OA into clinical care. Evidence-based treatment plan for OA, consisting of patient education and neuromuscular exercise	Approx. 10,000 patients/year in Denmark (2013–2020)	Coverage by universal healthcare: 100% reimbursement when referred by an orthopaedic surgeon; 40% coverage when referred by a general practitioner; 0% when self-referred (patient must pay all costs)
OA HWFL (31–34)	Australia	Improvement of daily living and associated quality of life by reducing knee and hip pain and stiffness and improving mobility. Furthermore, improvement of preparation (pre-hab) for knee or hip replacement surgery (if relevant).	Approx. 1,125 patients/year (2008–2016)	Coverage by universal healthcare: 100% of programme costs if participants eligible (i.e., over 548 USD worth of products, service and support, provided for free)
AMSOA (35–38)	The Netherlands	Collection of data (AMS-OA cohort): Provision of insight into the relation between clinical characteristics and functional outcome of patients with hip or knee OA.	Approx. 82 patients/year (2009–2017)	Coverage by universal healthcare: Full, partial, or no coverage (according to the health care policy and supplementary insurance)
JIGSAW (39–41)	United Kingdom (EU)	Primary care support: - addressing the unmet needs of adults with OA, through the provision of innovations - systematic implementation of International Guidelines and Quality Standards for OA at practise level across European sites	Primary Care in Clinical Commissioning Groups (CCGs) UK. 3 groups, ~100 practises	Coverage by universal healthcare: 100% by the National Health Service (NHS). European Institute of Innovation & Technology (EIT)-Health funded Joint Implementation of osteoarthritis Guidelines in Western Europe (JIGSAW-E). Implementation of this funded model was being tested in the Netherlands, Norway, Denmark and Portugal.

*OA, Osteoarthritis; Approx., Approximately*.

### Use of Framework for Programme Analysis

The RE-AIM framework was used to analyse the Reach, Effectiveness, Adoption, Implementation, and Maintenance of the six programmes (42). Briefly described, “the reach dimension of the framework refers to the percentage and characteristics of individuals receiving the intervention; effectiveness refers to the impact of the intervention, including anticipated as well as unanticipated outcomes; adoption concerns the percentage and representativeness of settings that adopt the intervention; implementation refers to the consistency and cost of delivering the intervention; and maintenance refers to long-term sustainability at both the setting and individual levels” (43).

The dimensions manifest themselves at various implementation levels (individual, organisation, community) and can facilitate the investigation of the impact of a programme on public health in a specific health care system (44). The RE-AIM dimensions were used to develop a survey to rate the applicability of each programme to the Swiss health care system. Glasgow et al. (15) stated that, “The use of the RE-AIM metrics might not result in a clear-cut decision, but it will facilitate the more informed and comprehensive consideration of all the relevant factors and make explicit the values and priorities” (42). The RE-AIM website (www.re-aim.org) defines a score of 9–10 as “excellent,” 7–8 as “good, but needs a little work,” 5–6 as “fair, needs additional attention,” and <5 as “poor, needs serious attention.”

### Development of Survey

The questions in the survey were based on the key pragmatic planning questions for RE-AIM formulated by Glasgow and Estabrooks (44). They were further developed for the survey to rate the applicability of the six chosen programmes for implementation in Switzerland (44). The original RE-AIM questions were used as headings for each dimension. The context of the programmes in each dimension was explained in a short text and the developed questions posed to rate the applicability. These questions are displayed in Table 2.

**Table 2:** Individual RE-AIM characteristics with the guiding questions for the analysis of the six programmes.

**BOA (8, 22, 23), Sweden**	**OACCP (25–27), Australia**	**GLA:D^®^ (7, 29), Denmark**	**OA HWFL (web-based program) (31–33), Australia**	**AMSOA (35–37), The Netherlands**	**JIGSAW (39), United Kingdom**
**“Reach”—Who is intended to benefit and who actually participates in the intervention?**					
Symptoms of hip, knee, or hand OA (referred or self-referred)	Diagnosed knee or hip OA with pain ≥4/10 (referred)	Symptoms of, or diagnosed hip or knee OA (referred or self-referred)	Overweight (BMI ≥ 28) patients with diagnosed (supported by X-Ray) hip and knee OA, for pre-rehab	Diagnosed hip or knee OA; ≥18 years; non-traumatic pain	GP consultation because of hip, knee, hand, foot OA, and joint pain; ≥45 years
**“Effectiveness”—What are the most important benefits (primary outcomes) you are trying to achieve and what is the likelihood of negative outcomes?**					
Pain, physical function, PA, QoL, and self-efficacy. Fear-avoidance behaviour and reported sick leave.	Pain, physical function, willingness for surgery, length of stay after surgery.	Pain, physical function, PA, QoL. Physical performance, self-efficacy. Use of painkillers, reported sick leave.	Weight loss, improved nutrition, QoL.	Pain, physical function, activity limitations (knee muscle strength), physical performance, and knee instability.	Uptake of Quality Indicators of NICE guidelines.
**“Adoption”—Where (in outpatient setting) will the programme be applied and who (provider/health care professionals) will apply it?**					
**Where:** Care centres	**Where:** Public hospitals, outpatient clinics	**Where:** Private practises and municipal rehabilitation centres	**Where:** Online and phone-based	**Where:** Rehabilitation centre	**Where:** Primary care practises
**Who:** PTs, OTs, expert patients	**Who**: Coordinated multidisciplinary team	**Who:** PTs and expert patients	**Who:** Coordinated care support team (pharmacists, dietitians, OTs, nurses)	**Who:** Coordinated multidisciplinary team	**Who:** GPs and practise nurses or PTs (depending on the country's primary care system)
**“Implementation”- How consistently can the programme be delivered, are adaptations needed (regarding content and duration)?**					
**Duration:** 12 or more weeks	**Duration**: 6–8 weeks	**Duration:** 8 weeks	**Duration:** 18 or more weeks (up to 2 years)	**Duration**: 12 weeks	**Duration**: Consultation model and additional offer of 4 sessions
**Content:** Consultation at multidisciplinary clinic. Individualised exercises sessions (twice a week) in groups or home-based: strength, cardiovascular training. Face-to-face progress reassessments.	**Content:** OA education session, 6-8 weeks group or home-based exercise sessions.	**Content**: 2 OA education sessions, 12 Individualised exercise sessions (60 min/twice a week) in groups or home-based: NEMEX.	**Content:** (telehealth) 4 dietetic consultations and lifestyle education. PA plan and PT-developed exercises (strength, balance and mobility exercises). 3 phases of minimum 6 weeks: Motivate, Consolidate, Maintain.	**Content:** 12 weeks of group exercise sessions (60 min): knee joint stabilisation, muscle strength, performance of daily activity; and home exercises (5 days/week). Supplementary: Psychological support and medical management.	**Content**: OA education, including OA guidebook. Advice on exercise and PA. Additional offer: Analgesia and referral to the practise nurse for 4 sessions to support self-management.
**“Maintenance”—When will the initiative become fully operational (system level), how long do results last (patient level), how long will the initiative be sustained?**					
**Patient level:** Follow-ups at 3 and 12 months	**Patient level:** Follow-ups at 12, 26, and 52 weeks	**Patient level:** Follow-ups at 3 and 12 months	**Patient level:** Follow-ups at 6 and 18 weeks, if longer duration: at least every 6 months	**Patient level:** Follow-ups at 6, 12, and 38 weeks	**Patient level:** No follow-up (consultation model)
**System level:** Operating nationwide. No referral needed for patients to participate.	**System level:** Operating nationwide. Included in different pathways, i.e., EMOS, a clinical pathway defined by orthopaedic surgeons.	**System level:** Operating nationwide.	**System level:** Included in national (and international) treatment guidelines for weight loss in OA.	**System level:** Operating in the rehabilitation centre where it was originally developed.	**System level:** Approximately 100 practises across 6 collaborating sites in 6 countries.

*OA, Osteoarthritis; PA, Physical activity; PT, Physiotherapist; OT, Occupational Therapist; GP, General Practitioner; EMOS, Enhanced Management of Orthopaedic Surgery*.

For each of these programme characteristics, the RE-AIM dimensions were rated on a Likert scale from 0 (not applicable at all) to 10 (very applicable). The programmes were anonymized, and their characteristics presented in a random sequence to prevent programme identification.

### Analysis of the Ratings

The overall mean scores with standard deviations (SD) of each programme and all dimensions were calculated and represented in a bar chart to provide a visual display. The programmes scoring >7 for the various dimensions were considered to be (highly) applicable for implementation in Switzerland.

## Results

### Analysis of the Programmes Using RE-AIM

Table 2 shows the programme characteristics, based on the RE-AIM dimensions, rated by the survey experts.

*Dimension “Reach*”: all the programmes included patients with knee pain and/or knee OA and provided an intervention based on exercise and patient education. OA HWFL only included patients with knee OA and/or knee pain and a BMI > 28, with the focus mainly on support for weight management. JIGSAW was the only programme that had no exercise sessions included, giving only advice on exercise and physical activity.

*Dimension “Effectiveness*”: the impact of individualised exercise and education on pain, function and quality of life was assessed for all programmes with follow-up and showed a significant effect in all those outcomes. JIGSAW, which had no follow-up, improved the patient pathway by applying the National Institute for Health and Care Excellence (NICE) guideline recommendations.

*Dimension “Adoption”*: all programmes were well-established in their country of origin, and some in other countries also. BOA, OACCP, GLA:D^®^, AMSOA, and JIGSAW were offered in outpatient settings, such as clinics, care or rehabilitation centres, and clinical practises. OACCP was provided in hospitals and outpatient clinics, AMSOA in a rehabilitation centre, and OA HWFL in an online and phone-based setting. Both AMSOA and OACCP combined numerous health care professions in multidisciplinary teams, whilst the other programmes only involved two to three health care professions.

*Dimension “Implementation”*: the programmes were similar in their content (e.g., support for self-management, patient education, and exercise) and in the minimal duration of the programmes, but their exercise programmes differed in intensity and the opportunity to prolong. The duration of the programmes was normally a minimum of 6 weeks and maximum of 3 months. The OACCP and OA-HWFL programmes from Australia offered additional longer-term support when necessary. The programmes varied in their approaches, but, at a minimum, all programmes included consultation or educational sessions, together with recommendations on exercises or an exercise programme. The structure of the JIGSAW programme was different and focused on the first consultation in primary care and on patient education. JIGSAW, a consultation model, included an additional four sessions of patient education, but with no follow-up measures for patients. The supervised and home-based exercise programmes accorded with international guidelines but were applied with different concepts.

*Dimension “Maintenance”*: The follow-ups to measure the outcomes of the patients were assessed after completion of the exercise sessions and a few months after the end of the programme. The operationalisation of the programmes was analysed on the system level. All programmes showed a nationwide roll-out and, therefore, good “Adoption” by the target population (which influences “Maintenance”). AMSOA was the only exception, since the programme remained well-established only in the rehabilitation centre where it was originally developed. The six programmes were initiated between 2008 and 2013 and are all ongoing.

### Rating of Applicability

**Figure 1:**
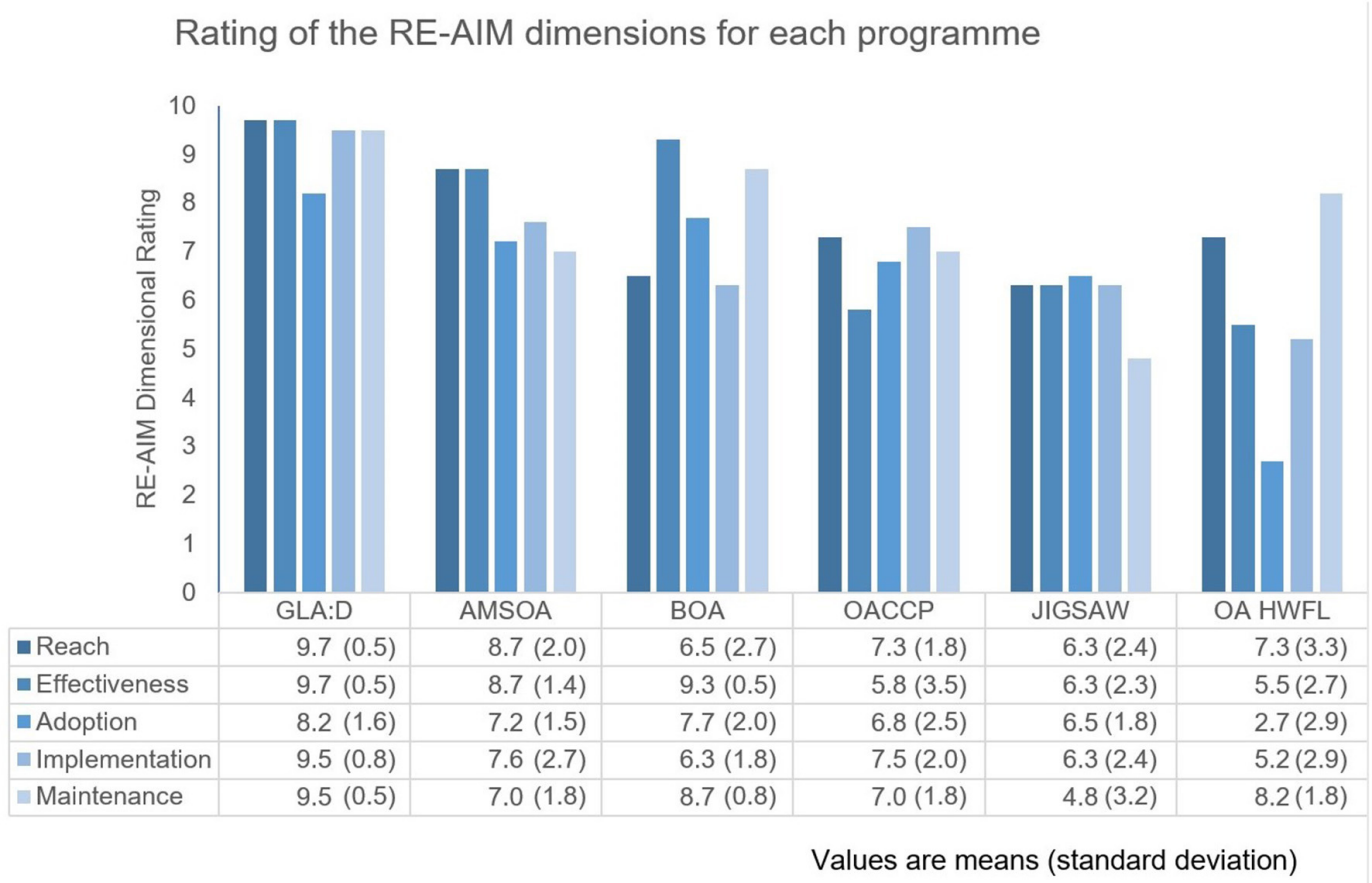
Ratings of the RE-AIM dimensions for each programme.

Six of the nine network members invited to participate (66.7%) returned their ratings. The clinical practitioners representing two different specialist physiotherapy societies and a representative of a German universality of applied Sciences did not respond. Figure 1 displays the mean scores of the ratings on the RE-AIM dimensions for each programme. The means scores across the six programmes ranged between 5.9 and 9.4. The overall mean scores of each programme and the range of the mean scores of dimensions were: GLAD 9.4 (8.2–9.7), AMSOA 7.9 (7.0–8.7), BOA 7.8 (6.3–9.3), OACCP 7.0 (5.8–7.5), JIGSAW 6.3 (4.8–6.5), and OA HWFL 5.9 (2.7–8.2).

## Discussion

The comparative analysis and rating of the six programmes, following the RE-AIM framework, found that the GLA:D^®^ programme would be the most applicable to the Swiss health care system. The result suggests that GLA:D^®^ could be implemented successfully in Switzerland with only few adaptations.

### Implementation Considerations

The ratings provide guidance for planning both the implementation strategy and the activities on the different dimensions. Through consideration of the respective levels, the results of this rating assist in deciding which dimensions need more attention during the development of the implementation strategy and activities. For example, combining the locations (where) and the health care professionals involved (who) resulted in the lowest scores in “Adoption” for all programmes. In the complex and decentralised Swiss health care system, together with the high autonomy of the health care professions, an innovation would have a better chance of successful implementation when only a few key stakeholders were involved in the programme. Furthermore, an evidence-performance gap is present in the conservative management of knee OA, thus the programme is intended to be offered in the outpatient setting. GLA:D^®^ is offered mainly in outpatient settings and only involves a few stakeholders. Therefore, the programme reached a relatively high score of 8.2, meaning “good, but needs a little work.”

So, the team implementing GLA:D^®^ would need to pay extra attention to “Adoption” during the implementation process. “Adoption” is associated with the leading indicators of acceptability, appropriateness, and feasibility (45). The implementation activities should, therefore, focus on those leading indicators and involve important stakeholders to maximise scores in “Adoption.” The standardisation of the outcome measures and reporting could improve interdisciplinary work between referring doctors and physiotherapists. However, OACCP stated that it was essential that all the health care professionals involved were convinced that the programme enhances conservative management (10).

In the other four dimensions, i.e., Reach, Effectiveness, Implementation, and Maintenance, GLA:D^®^ showed a mean score of 9.5 or higher, which is defined as “excellent.” These dimensions would require fewer adaptations and need less attention during the implementation process. This means that targeting patients with knee OA or knee pain, with no further inclusion or exclusion criteria, seems to be a good option, according to the rating. Other programmes reached lower scores because they set an age limit, or only include referred patients, or overweight patients with a BMI ≥ 28. High scores for GLA:D^®^ also resulted from its main focus being on the symptoms of knee OA, physical function, and the behaviour of the patients (in terms of fear-avoidance behaviour or sick leave). The additional factors in other programmes (e.g., willingness for surgery, weight control, nutrition), which are not intended to be included in the primary outcomes of a Swiss programme, might have resulted in lower programme scores. Regarding the content, the four programmes BOA, AMSOA, GLAD, and OACCP provide exercise sessions with supervision. Individualised and supervised exercise is applied to ensure sufficient load and progression and to support quality of performance for long-term self-management (46). Supervised exercise may improve adherence and may also lead to better outcomes (22). GLA:D^®^ integrates neuromuscular exercises (NEMEX), i.e., functional knee stability, during the exercises. NEMEX have been proven to be safe and effective (32, 46). Sensomotor control and functional stability should be supervised by a physiotherapist, because the quality of performance, i.e., in NEMEX, is crucial (46). The four programmes combine supervised with home-based exercises for best results (46). The high score (9.5) of GLA:D^®^ for “Implementation,” with a difference of almost 2 compared to the other programme scores, indicates that the duration of 8 weeks, or 12 sessions, and the integration of NEMEX might be well-accepted by all parties, i.e., patients, providers, and insurers. In contrast, the costs of online treatments, or a longer duration, e.g., OA HWFL, might not be fully covered by Swiss insurers. High scores for “Maintenance” were reached in programmes with follow-ups at 3 and 12 months, together with the strategy for a nationwide rollout with no referral needed for participation. This seems to be the “Maintenance” strategy most compatible with the intended goals of a Swiss programme.

### Additional Offers for Improving Conservative Management

This analysis and the ratings show that also the lower scoring programmes, i.e., all the programmes except GLA:D^®^, have some important aspects that impact the implementation goals in Switzerland.

### Online Programme

After the initial implementation of a programme, some other aspects need to be considered for potential inclusion, e.g., in times of a global pandemic, an online programme has the advantage that patients can perform the exercises whenever, or wherever, they want. BOA provides an additional web-based version, named “Joint Academy” (47). Although direct support from a physiotherapist showing how to perform the exercises would be lacking, some patients might prefer an online programme. Joint Academy offers online support from a physiotherapist for 6 weeks duration. Personalised exercises are delivered by email and supported by videos and take-home messages. Although an online exercise programme might not be supported financially by Swiss healthcare insurers, patients may choose to pay themselves for this type of offer.

### Weight Management

OA HWFL, which focused on weight reduction and nutrition, achieved lower scores for applicability for implementation in the Swiss health care system in most RE-Aim-dimensions. However, the working collaboration of referring medical doctors, physiotherapists, and nutritionists helped to improve knee OA management (48). Overweight is a risk factor in developing knee OA and has a negative impact on the course of this chronic disease. Weight reduction improves knee pain and function (49, 50). Weight management could, therefore, be beneficial and should be considered when the population shows many cases of knee OA correlating to overweight or obesity. This additional intervention, ideally complementary to an exercise and education programme, also has the potential to improve knee OA management (31).

### Written Information

The JIGSAW programme had the lowest mean score over all dimensions for applicability for implementation in the Swiss health care system, compared to the other programmes. Although patient education is included in the exercise and education programmes of, e.g., GLA:D^®^, BOA, or AMSOA, a written booklet elaborating the most important information on OA could further support patient self-management or motivate them to join an exercise and education programme.

### Strengths and Limitations

A strength of this study is the use of the RE-AIM dimensions to analyse and rate the chosen programmes to ascertain which would be most applicable for implementation in Switzerland, and to indicate potential challenges to the implementation process. A limitation of this analysis could be selection bias, since the information provided in the survey was the only information on the programmes that was either published in English or available on the respective websites. Another limitation may be that the non-responders included the two different specialist physiotherapy societies could therefore lead to underrepresentation of clinical practitioners, even though there was still one participant who is a researcher but also working in clinical practise. The underrepresentation could result in a lack of the opinions or perspectives of people who are specialised in the practical application of a programme, especially, on the feasibility of the contents, (i.e., dimension implementation) or the practicability of the programme when there are many different health care professionals included (i.e., dimension adoption). However, this study focused on the representatives of the main providers of such a programme in Switzerland, i.e., physiotherapists, who are important stakeholders in the future implementation of an exercise and education programme for the management of knee OA.

In Switzerland, there is a need for a structured and systematic programme for patients with knee OA, due to the high prevalence of the disease and the lack of knowledge of the beneficial effects of exercise and education. A renowned and established programme might be favourably accepted and contribute to closing the existing evidence-performance gap in clinical practise. Furthermore, such a programme would represent an improvement in non-surgical and non-pharmacological management and follow-up. In conclusion, the GLA:D^®^ programme with the highest scores has already been implemented in other countries. The programme consists of exercise and education and scored higher than 7 in all RE-AIM dimensions. Therefore, this programme is most applicable to the Swiss health care system as only few adaptations would be needed for its successful implementation in Switzerland.

## Data Availability Statement

The raw data supporting the conclusions of this article will be made available by the authors, without undue reservation.

## Ethics Statement

This study did not fall within the scope of the Swiss Human Research Act and authorisation from an Ethics Committee was not required. Raters were informed that by participation in the survey they automatically provided their informed consent and that their ratings were used in an anonymised manner.

## Author Contributions

LE and KN were contributing to conception and design of the study. LE collected and analysed the programme information and data of the ratings. KN contributed to the drafting and revision of the manuscript. All authors have read and approved the manuscript.

## Conflict of Interest

KN is a member of the network of experts in physiotherapy for knee OA management. However, KN did not participate in the survey and the ratings. The remaining authors declare that the research was conducted in the absence of any commercial or financial relationships that could be construed as a potential conflict of interest.

## Publisher's Note

All claims expressed in this article are solely those of the authors and do not necessarily represent those of their affiliated organizations, or those of the publisher, the editors and the reviewers. Any product that may be evaluated in this article, or claim that may be made by its manufacturer, is not guaranteed or endorsed by the publisher.
